# Severe methemoglobinemia secondary to isobutyl nitrite toxicity: the case of the ‘Gold Rush’

**DOI:** 10.1093/omcr/omaa136

**Published:** 2021-02-15

**Authors:** Gregory M Taylor, Robert S Avera, Christian C Strachan, Christian M Briggs, Jason P Medler, Carl M Pafford, Timothy B Gant

**Affiliations:** 1 Assistant Professor of Clinical Emergency Medicine, Indiana University School of Medicine; IU Health Ball Memorial Hospital, Muncie, IN, USA; 2 Toxicology Fellow, Indiana University School of Medicine, Indianapolis, IN, USA; 3 Executive Vice Chair of Clinical Affairs, Assistant Professor of Clinical Emergency Medicine, Indiana University School of Medicine; East Central Region Emergency Department Medical Director, Indianapolis, IN, USA; 4 Resident, Indiana University School of Medicine; IU Health Ball Memorial Hospital, Muncie, IN, USA; 5 Emergency Department Nurse; IU Health Ball Memorial Hospital, Muncie, IN, USA

## Abstract

Isobutyl nitrite is one of the popular recreational drugs with high abuse potential that is known to cause methemoglobinemia. While inhaling this recreational drug, often referred to as a ‘popper’, is the typical route of administration, oral ingestion can produce a more rapid and fulminant course of methemoglobinemia. We present the case of a 69-year-old male that presented to our emergency department in extreme, life-threatening methemoglobinemia due to the ingestion of isobutyl nitrite that he obtained from an adult novelty store. The patient had a methemoglobin level above our lab cut-off of 28% and was subsequently treated with two doses of intravenous methylene blue. His hospital course was unremarkable, and he was discharged on Day 2. Methemoglobinemia is a medical emergency that requires a high index of clinical suspicion, prompt recognition, and rapid treatment.

## INTRODUCTION

The inhalants are often marketed in glass-vials as a nail-polish remover. They are often found in adult stores under nicknames like ‘Rush’, ‘Bolt’, and ‘Jungle Juice’ [1]. These agents are readily absorbed on virtually any body surface [2]. Hypotension and reflex tachycardia can occur within 30 s of inhaling just five drops [3]. Other side effects include headache, facial flushing, dizziness, confusion, tracheobronchitis and methemoglobinemia [3].

## CASE REPORT

A 69-year-old male with a significant past medical history of hypertension presented to the emergency department (ED) via EMS for reported hypotension and syncope. The patient was picked up in an adult novelty store after employees noted he kept falling, looked ill and finally collapsed to the floor shortly after checkout. The patient was not forthcoming of information and denied ingesting anything. His only complaints were dizziness, headache and fatigue.

Vitals on arrival to the ED: hypothermic at 34.7°C, heart rate 106 beats/min, respiratory rate 32 breaths/min, blood pressure 72/40 mmHg and pulse oximetry of 85% on room air. On physical exam he appeared ill and cyanotic, however, he was alert and oriented ×3 and was moving all extremities. His skin appeared to have a gray discoloration. Capillary refill was >3 s. The remaining physical exam was unremarkable.

**Figure 1:**
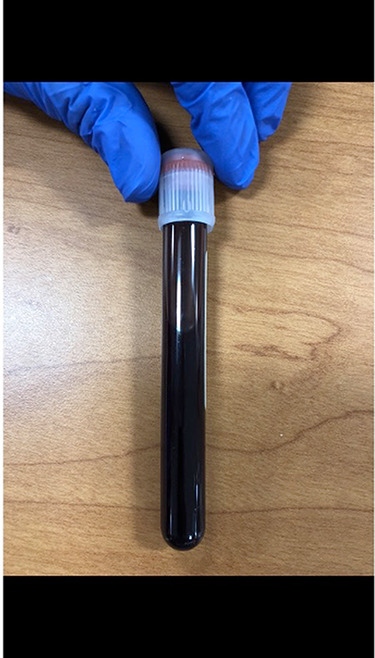
Tube of blood that appears chocolate colored.

**Figure 2:**
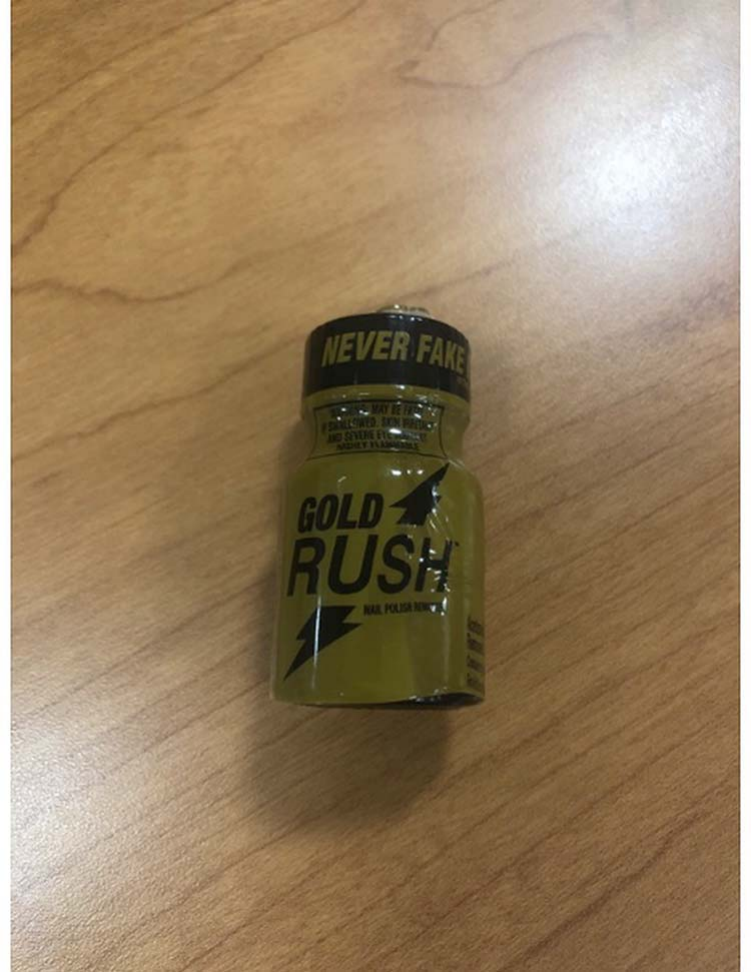
‘Gold Rush,’ marketed as a nail polish remover. It contained 9 ml of isobutyl nitrite.

We noted the tube of blood, Fig. 1, appeared chocolate colored. Despite 30 cc/kg of fluid resuscitation, his blood pressure remained 82/47 mmHg and norepinephrine was started. In addition, his pulse oximetry did not change despite being placed on a non-rebreather, and would intermittingly show a pulse oximetry of 62%, then 82% and appeared to constantly be fluctuating. While staff was helping the patient into a gown, a bottle of ‘Gold Rush’, Fig. 2, was discovered that contained 9 ml of isobutyl nitrite. The patient admitted that he bought one and thought he was supposed to drink it as he had planned some intimate activities with his wife. When he did not see any results, he quickly went back to the adult store and bought a second one. He managed to pay for it prior to collapsing.

His electrocardiogram revealed sinus tachycardia at 110 with inverted T-waves in lead II and III, with ST depressions in V1-V3. His complete metabolic panel was notable for a creatinine of 1.5 mg/dl, lactic acid of 2.9 mmol/L, and an ethanol level of 159. An arterial blood gas revealed a pH of 7.26, pCO2 of 40, pO2 of 88, bicarb of 17.9 and an oxygen saturation of 95%. A serum methemoglobin quantitative level (Bld QN) was >28% (our lab cut-off). His remaining labs, imaging and toxicology screen were negative.

We discussed the case with the Indiana Poison Control Center and began treatment with intravenous methylene blue at 2 mg/kg over 15 min. His cyanosis remained after 30 min and he was given a second dose of 2 mg/kg over 15 min. He was subsequently admitted to the intensive care unit (ICU). All his symptoms resolved by 7-h post methylene blue infusion with a repeat methemoglobin level at ~14 h of 1.6%. His remaining hospital course was unremarkable, and he was discharged the next day.

## DISCUSSION

Under normal circumstances, healthy patients have <1% of methemoglobin [4]. In methemoglobinemia, there is an elevation of circulating methemoglobin in the blood. It is produced when iron in the hemoglobin molecule is in the ferric state (Fe3+), as compared to the ferrous state (Fe2+). As a result, this oxidized form of hemoglobin has decreased oxygen and carbon dioxide carrying capacity, and decreased oxygen deposition to tissues [5].

While methemoglobinemia can be both inherited and acquired, it is most commonly caused by exposure to medical substances, exhaust fumes, herbicides/pesticides, and chemicals [4]. Overall, most cases of acquired methemoglobinemia result from exposure to medications. Some of the most common medications are found in Table 1 [5, 6]. Amyl nitrate and isobutyl nitrite are popular recreational drugs used for both the vasodilatory effect and euphoric effects, making them an inhalant with a high-abuse potential [7]. The vasodilation and reflex tachycardia can create a feeling of enhanced sexual pleasure and euphoria [7]. Evidence shows that the response to methylene blue is relatively quick and may only require a second bolus in severe cases [5].

**Table 1:** Some of the most common causes of acquired methemoglobinemia

Aniline	Benzocaine derivatives	Bupivacaine	Nitrates/nitrites
Nitric oxides	Sodium nitroprusside	Nitroglycerine	Phenazopyridine hydrochloride
Phenytoin	Rifampin		

Patients that present to the ED as an acquired methemoglobinemia can present with a myriad of complaints and can follow rapidly fatal course. This is particularly true in cases in which an accurate and reliable history is unavailable. A diagnosis is based on the results of an arterial blood gas and concentration of methemoglobin in the blood [4]. The clinical effects of methemoglobinemia are mainly dependent on the quantity of methemoglobin (MetHb), which is typically reported as a percentage of the patient’s total hemoglobin [8]. Clinicians often see a sudden onset of cyanosis that does not improve with oxygen administration in addition to a low pulse oximetry reading. It should also be suspected in those patients that appear cyanotic, however, have a normal arterial pO2 [5]. It should be noted that pulse oximetry is often very inaccurate and should not be relied on, as standard pulse oximetry only records levels of oxyhemoglobin/deoxyhemoglobin [7].

When the MetHb level approaches 1.5 g/dl, patients will appear cyanotic [9]. This is equivalent to ~10% in patients with normal levels of hemoglobin [10]. At 30%, this is considered life-threatening [5]. Table 2 is taken from the Indiana Poison Center treatment guidelines as it pertains to the clinical presentation based on the percentage of MetHb [8].

**Table 2:** Signs and symptoms based on the concentration of MetHb

MetHb (%)	Signs and symptoms
3–20	Low pulse oximeter readings, slate gray skin discoloration, chocolate brown blood and cyanosis
20–50	Headache, syncope, dizziness, weakness, fatigue and dyspnea
50–70	Tachypnea, CNS depression, metabolic acidosis, seizures, dysrhythmias and coma
>70	Severe hypoxic damage and death

The first-line treatment is methylene blue. Methylene blue is an oxidizing agent, which in the presence of NADPH and NADPH Methemoglobin Reductase is converted to the active form leukomethylene blue [9]. Leukomethylene blue acts by regenerating the ferrous heme back from the ferric state via the NADPH reductase pathway [7]. Indications for treatment with methylene blue includes symptomatic patients with a MetHb > 30%. Clinicians can consider administration if the patient is symptomatic with a MetHb > 20% [8]. It should be noted that patients with pre-existing cardiorespiratory problems or anemia should be treated at lower levels of MetHb [6]. Other management modalities reported include an exchange transfusion and ascorbic acid in those with a G6PD deficiency, hyperbaric O2 when intravenous methylene blue is contraindicated or ineffective, in addition to bicarbonate and intravenous hydration for metabolic acidosis.

A recommended treatment regimen, taken from the Indiana Poison Center treatment guidelines, includes an initial IV bolus of 1–2 mg/kg given over several minutes. If the patient’s cyanosis has not disappeared within 30–60 min, a second dose of 1–2 mg/kg can be administered [8]. If there is no resolution after the second bolus, the clinician should consult with their toxicologist again as alternative therapies for refractory MetHb exist, including in those patients with G6PD deficiency. There are published cases in the literature of severe methemoglobinemia from inhaling isobutyl nitrite, however, severe cases involving oral ingestion of isobutyl nitrite are rarely reported.

## Data Availability

Data sharing is not applicable to this article as no datasets were generated or analyzed during the current study.
